# Intra- and interspecific toxicity testing methods and data for nematodes exposed to metals

**DOI:** 10.1016/j.dib.2021.107544

**Published:** 2021-11-06

**Authors:** Scott Glaberman, Andrew Heaton, Scott Weir

**Affiliations:** a Department of Environmental Science & Policy, George Mason University, Fairfax, VA 22030, USA; b Grand Bay National Estuarine Research Reserve, Moss Point, MS, USA; c Department of Biology, Queens University of Charlotte, Charlotte, NC, USA

**Keywords:** C. elegans, Toxicology, Copper, Zinc

## Abstract

Twenty-four hour median lethal concentration (LC50) toxicity tests were performed with five species of nematodes (*Caenorhabditis elegans, Caenorhabditis briggsae, Pristionchus pacificus, Oscheius tipulae*, and *Oscheius myriophila*) in response to copper chloride and zinc chloride. In addition, lethality tests were also performed with seven strains of *C. elegans* (N2 > 1 year in culture, N2 newly acquired, N2 ancestral, ED3053, JU258, JU1171, and MY1) exposed to copper chloride. Nominal chemical concentrations were validated and analyzed according to U.S. Environmental Protection Agency method 6010 using inductively coupled plasma–atomic emission spectroscopy (ICP-AES). This paper combines the datasets previously published separately by Heaton et al. (2020, 2022). The goal is to catalog all raw and analyzed toxicity data collected from both studies in a single consistent information source for use by the scientific community.

## Specifications Table

**Table:** 

Subject	Health, Toxicology and Mutagenesis
Specific subject area	Environmental Toxicology
Type of data	Table(s)
How data were acquired	Nematode toxicity tests were performed using a standard stereomicroscope and visual observation. Copper and zinc concentrations were validated using an inductively coupled plasma–atomic emission spectrometer (ICP-AES) according to EPA Method 6010.
Data format	Raw, Analyzed
Description of data collection	24 h toxicity tests with nematodes were carried out in multi-well plates containing copper, zinc, or K-medium controls. LC50 values for 5 nematode species and seven *C. elegans* strains were determined by counting the number of live/dead nematodes after 24 hours of exposure.
Data source location	Institution: University of South Alabama City/Town/Region: Mobile, Alabama Country: USA
Data accessibility	Repository name: Mendeley Direct URL to data: http://dx.doi.org/10.17632/v2wzhr3twmhttps://data.mendeley.com/datasets/v2wzhr3twm
Related research article	Heaton A, Milligan E, Faulconer E, Allen A, Nguyen T, Weir S, Glaberman S. Variation in copper sensitivity between laboratory and wild strains of Caenorhabditis elegans. Chemosphere. 2022 https://doi.org/10.1016/j.chemosphere.2021.131883
	Heaton A, Faulconer E, Milligan E, Kroetz MB, Weir SM, Glaberman S. Interspecific variation in nematode responses to metals. Environmental toxicology and chemistry. 2020 May;39(5):1006–16. https://doi.org/10.1002/etc.4689

**Value of the Data**

• Provides raw and analyzed nematode toxicity data that can be combined with future datasets from other nematode strains/species or chemicals generated by other researchers.
• Researchers working on metal toxicity or ecotoxicology of nematodes can use these data to build intra- or interspecific toxicity models. Ecological risk assessors can use these data to better understand/account for variability in species sensitivity to contaminants.

## 1 Data Description

**File 1 - Nematode Raw Toxicity Data.xlsx** – Contains raw toxicity trial data for lethality tests with copper chloride and zinc chloride with nematodes. Copper chloride was tested on 5 nematode species and 7 *C. elegans* strains. Zinc chloride was tested on 5 nematode species.

**File 2 - Nematode LC50 Calculation Tables.xlsx** – Contains LC50 calculations for all nematode lethality tests with copper chloride and zinc chloride. LC50 values, standard errors, and 95% confidence intervals were calculated for each experimental trial with each species/strain as well as for combined trials for each species/strain. LC50 calculations for copper were based on nominal copper ion concentrations. LC50 calculations for zinc were based on verified zinc ion concentrations.

## 2 Experimental Design, Materials and Methods

**Overview:** Twenty-four hour median lethal concentration (24hr-LC50) toxicity tests were performed with 5 species of nematodes (*Caenorhabditis elegans, Caenorhabditis briggsae, Pristionchus pacificus, Oscheius tipulae*, and *Oscheius myriophila*) in response to copper chloride and zinc chloride. In addition, lethality tests were also performed with 7 strains of *C. elegans* (N2 >1 year in culture, N2 newly acquired, N2 ancestral, ED3053, JU258, JU1171, and MY1) exposed to copper chloride. These data were originally published separately by Heaton et al. [1,2]. Both raw and analyzed toxicity data are reported here to catalog all data collected from both studies into a single consistent information source for use by the scientific community.

**Chemical source and analysis:** Cupric chloride (99% purity, Acros Organics) and zinc chloride (>98% purity, Alfa Aesar) were used to prepare test material. A subset of nominal test concentrations were analytically verified using a commercial laboratory testing service (Pace Analytical Services, St. Rose, Louisiana) as reported in Heaton et al. [2]. Samples were analyzed according to EPA Method 6010 using Inductively Coupled Plasma (ICP) – Atomic Emission Spectroscopy. The reporting limit ranged from 0.05 to 1 mg/L across samples and was always at least one order of magnitude below the target nominal concentration. Since copper verified concentrations were > 90% of nominal concentrations, we used nominal copper ion concentrations for copper LC50 calculations. Conversely, since zinc concentrations were consistently 70% of nominal, we used verified zinc ion concentrations for all zinc LC50 calculations. For zinc concentrations that were not tested, we used the regression formula in Heaton et al. [2] to correct nominal concentrations for the expected verified concentrations.

***Nematode cultures*:** One strain each of *Caenorhabditis briggsae* (AF16), *Pristionchus pacificus* (PS312), *Oscheius tipulae* (CEW1), and *Oscheius myriophila* (EM435), and six strains of *C. elegans* (N2, ancestral N2, ED3053, JU258, JU1171, MY1) were obtained from the Caenorhabditis Genetics Center (CGC) at the University of Minnesota. An additional N2 strain in culture for >1 year was obtained from the laboratory of Mary B. Kroetz at the University of South Alabama.

All strains were maintained on solid support media (NGM agar) with a lawn of *E. coli* strain OP50. NGM plates and E. coli lawns were prepared using methods adapted from Stiernagle [3]. To prepare NGM plates, 3 g NaCl, 17 g agar, 2.5 g peptone, and 975 ml H_2_O were added to a 2 L Erlenmeyer flask. The mouth of the flask was then covered with aluminum foil and autoclaved for 50 min. After autoclaving, the Erlenmeyer flask was cooled in a 55 °C water bath for 15 min, and the media was supplemented with 1 ml of 1 M CaCl_2_, 1 ml of 5 mg/ml cholesterol in ethanol, 1 ml of 1 M MgSO_4_, and 25 ml of 1 M KPO_4_ buffer and swirled to mix. Using sterile technique, the NGM solution was dispensed into petri dishes using a peristaltic pump, filling the plates so that they were 2/3 full of agar. The prepared plates were then left at room temperature for 2-3 days before use to allow for detection of contaminants, and to allow excess moisture to evaporate.

To prepare an OP50 bacterial lawn for feeding worms during testing, a starter culture was obtained from the CGC. Generally, when there were previously made NGM plates in the lab, OP50 was recovered from a plate using a flame-sterilized wire pick to start a new culture. L broth was prepared by adding 10 g Bacto-tryptone, 5 g Bacto-yeast, and 5 g NaCl to a 2 L Erlenmeyer flask. The flask was then filled to 1 L with reverse osmosis (RO) water, and then 1 M NaOH was used to obtain a pH of 7.0. The solution was dispensed into 250 ml screw-cap bottles (adding 100 ml per bottle) as needed and autoclaved. The prepared media was stored at room temperature for up to several months. After cooling to room temperature, the prepared broth was aseptically inoculated with the OP50 culture obtained from the CGC or from a prior NGM plate and allowed to grow overnight at 37 °C. The *E. coli* solution was then used to seed NGM plates using disposable pipet tips or glass pipettes using a streaking motion, and plates were refrigerated at 4 °C until use.

*C. briggsae, P. pacificus, O. myriophila, O. tipulae*, and multiple strains of *C. elegans* were all routinely cultured at 15 °C. All species except for *O. tipulae* were “fed” once per week by transferring a subset of 3–4 L4/J4 individuals to fresh plates with OP50 bacteria. Due to the difficulty in accumulating large numbers of *O. tipulae* for testing, this species had to be kept on larger 90 mm plates and transferred to new plates weekly using the chunking method to accommodate the larger number of worms needed for transferring. To chunk a plate, a flame-sterilized scalpel blade was used to slice a small triangular portion of agar containing worms/eggs from the edge of a week-old NGM plate. This chunk of agar is then placed with the eggs/worms touching the agar surface of a new NGM plate, and the worms resulting from these eggs provide the following weeks’ culture on the new plate.

In order to age-synchronize animals for toxicity testing, nematodes from eight-day-old plates were subjected to a bleaching process adapted from Stiernagle [3] in which gravid adults were removed leaving only eggs. Nematodes were prepared for bleaching first by washing them from NGM plates into 15 mL conical tubes and spinning in a swinging bucket centrifuge for 90 s to obtain a nematode pellet. After aspirating the supernatant, a bleach solution was added to the pellet, and the tube(s) containing the bleach solution/pellet were shaken vigorously by hand for 5 min. After shaking, the centrifugation/aspiration process was repeated 4 times with K medium to wash the bleach solution from the eggs. After aspirating the last supernatant, the resulting eggs were placed on NGM agar plates seeded with OP50 and kept at 20 °C. Three-day-old age synchronized worms were then used for transferring via flame-sterilized wire pick into test chemical and control wells.

**Toxicity tests:** Dilution series for toxicity tests with copper chloride and/or zinc chloride were made with complete K medium (1 L K medium, 1 mL cholesterol [5 mg/mL], 1 mL 1 M CaCl2, and 1 mL 1 M MgSO4). Initial range-finding tests were carried out for both chemicals and all species/strains at concentrations of 0, 1, 10, 100, and 1000 mg/L in 24-well culture plates containing 0.5 mL of test solution and 5 nematodes per well. Nematodes were not fed during mortality testing to avoid confounding interactions of OP50 bacteria with the test substance or test organism. Culture plates were placed on an orbital shaker (Hoefer Red Rotor at setting “4”) in a temperature controlled room at 20 °C for 24 ± 0.5 h. A 24 h exposure period was chosen because animals were not fed during the toxicity test and longer periods of exposure would have resulted in starvation of the nematodes. Individuals were counted as alive if moving or dead if they did not move in response to repeated probing with a wire pick.

Definitive LC50 toxicity test concentrations were based on the results from the range-finding study and utilized a spacing factor of 2. All tests included a negative K-medium control. For each test concentration, there were 4 replicates of 10 nematodes (40 nematodes total) each placed in a single well with 0.5 mL of test solution. For all tests, the concentration scheme was designed when feasible to obtain one concentration with no mortality, one concentration with complete mortality, and at least one concentration above and below 50% mortality. At least two definitive LC50 tests were conducted with each species, but additional test replicates were added if there was a visual lack of consistency in results between the first two tests. Raw toxicity data are reported in **File 1**. These raw data represent the entire datasets produced by Heaton et al. [1,2] in a single spreadsheet. The rationale for presenting all the raw data here is so other researchers can easily perform a reanalysis or compare directly with other toxicity datasets.

**Toxicity data analysis**: All dose-response models and statistical analyses were performed in R [4] using the “glm” function in the “MASS” package as described in Heaton et al. [1,2]. LC50 values, standard errors, and 95% confidence intervals were calculated for individual study trials and for all trials combined for each species/strain. The only exception was that two trials for copper (one from *P. pacificus* and one from *C. briggsae*) were removed from the analysis because their results differed significantly from the other trials, which was previously attributed to the development of our husbandry and testing protocols [2]. LC50 calculations for individual and combined trials are reported in **File 2**.

## Ethics Statement

This work did not involve any experimentation on vertebrate animals.

## CRediT authorship contribution statement

**Scott Glaberman:** Conceptualization, Methodology, Formal analysis, Writing – original draft, Supervision. **Andrew Heaton:** Investigation, Writing – review & editing. **Scott Weir:** Formal analysis, Software.

## Declaration of Competing Interest

The authors declare that they have no known competing financial interests or personal relationships that have or could be perceived to have influenced the work reported in this article.
